# Edward Stock, M.R.C.S.

**Published:** 1901-12

**Authors:** 


### EDWARD STOCK, M.R.C.S.

It has been almost forgotten that this venerable member of an old Bristol family was in any way connected with the medical profession. From the *Bristol Times and Mirror* of November 20th we learn that Mr. Stock became a Member of the Royal College of Surgeons of England in 1841, and that he afterwards did good work in battling an epidemic of cholera. Since that time he has held many civic offices in Bristol, but is chiefly remembered as the Secretary of the Bristol Dispensary, an office which he held for about thirty years. He was also the first Secretary and Registrar of the Bristol University College. Very early in life he developed symptoms of phthisis, and for many years has been suffering from indications of cardiac weakness; nevertheless he survived to the age of 84, and died at Clevedon on November 18th.

